# Antibiotic Resistance of *Legionella pneumophila* in Clinical and Water Isolates—A Systematic Review

**DOI:** 10.3390/ijerph17165809

**Published:** 2020-08-11

**Authors:** Olga Pappa, Dimosthenis Chochlakis, Vassilios Sandalakis, Chrysa Dioli, Anna Psaroulaki, Athena Mavridou

**Affiliations:** 1 Department of Biomedical Sciences, University of West Attica, 12243 Egaleo, Greece or o.pappa@eody.gov.gr (O.P.); chrysrose57@gmail.com (C.D.); 2 Central Public Health Laboratory, National Public Health Organization, EL-15123 Athens, Greece; 3 Laboratory of Clinical Microbiology and Microbial Pathogenesis, School of Medicine, University of Crete, 71500 Heraklion, Crete, Greece; surreydimos@hotmail.com (D.C.); v.sandalakis@uoc.gr (V.S.); psaroulaki@uoc.gr (A.P.)

**Keywords:** *Legionella pneumophila*, antibiotic resistance, water sample, clinical sample, antibiotics

## Abstract

The current systematic review investigates the antibiotic susceptibility pattern of *Legionella pneumophila* isolates from the 1980s to the present day, deriving data from clinical and/or water samples from studies carried out all over the world. Eighty-nine papers meeting the inclusion criteria, i.e., “*Legionella pneumophila*” and “resistance to antibiotics”, were evaluated according to pre-defined validity criteria. Sixty articles referred to clinical isolates, and 18 articles reported water-related *L. pneumophila* isolates, while 11 articles included both clinical and water isolates. Several methods have been proposed as suitable for the determination of MICs, such as the E-test, broth and agar dilution, and disk diffusion methods, in vivo and in vitro, using various media. The E-test method proposed by the European Society of Clinical Microbiology and Infectious Diseases (EUCAST) seems to be the second most frequently used method overall, but it is the preferred method in the most recent publications (2000–2019) for the interpretation criteria. Erythromycin has been proved to be the preference for resistance testing over the years. However, in the last 19 years, the antibiotics ciprofloxacin (CIP), erythromycin (ERM), levofloxacin (LEV) and azithromycin (AZM) were the ones that saw an increase in their use. A decrease in the sensitivity to antibiotics was identified in approximately half of the reviewed articles.

## 1. Introduction

Antimicrobial resistance in bacterial pathogens (ARBs) is a worldwide challenge associated with high morbidity and mortality. The extraordinary genetic capacities of microbes have benefitted from man’s overuse of antibiotics to exploit every source of resistance genes [1]. Multidrug-resistant patterns in Gram-positive and -negative bacteria have resulted in infections difficult to treat or even untreatable with conventional antimicrobials. Dramatic increases in emerging resistance occur, particularly when poor infection-control practices are coupled with resistant bacteria that can easily be disseminated to other patients and the environment. Globally, antimicrobial-resistant infections kill at least 700,000 people each year. Within 30 years, resistant microorganisms are predicted to kill 10,000,000 per year, greatly exceeding deaths from cancer [2]. The geographical distribution of resistance may occur, in part, due to medical practices but also due to practices in the agriculture, fisheries and meat industry of each country [3]. For instance, in a study testing sludges in various areas in China, a redundancy analysis model plot of the bacterial community composition clearly demonstrated that sludge samples differ significantly according to geographical area. The shifts in bacterial community composition were correlated with antibiotic resistance genes (ARGs) [4]. The abundance and type of ARGs may also differ between cities. In a study, the presence of *bla*_TEM_ and *str*B is an example of this problem [5].

An increasing awareness of the antibiotic-resistance crisis related to drinking water consumption seems to be growing in the scientific community, even though the number of relevant papers in the literature is not high. Antimicrobial resistance in bacterial pathogens (ARBs) of water microbiomes and ARGs in drinking water pose a significant public health concern when they transfer antibiotic resistance to human pathogens, a situation which is still unclear [6]. The effects of antibiotics on the microorganisms in the biofilms created in drinking water distribution systems (DWDSs) have also been studied [7], with *Pseudomonas aeruginosa* being the bacterium that has been approached as a priority [8].

*Legionella pneumophila* is a facultative intracellular pathogen known to cause community-acquired pneumonia (CAP). Monitoring the emerging issue of the antibiotic resistance of *L. pneumophila* isolates is a recent issue in the treatment of Legionnaires’ disease (LD) [9]. The antibacterial scheme used for legionellosis has been altered significantly over the years. Following the first outbreak of LD that occurred in 1976 among American Legionnaires in Philadelphia, erythromycin was proposed as the antibiotic of choice for the treatment of LD [10], which has changed significantly in the coming decades as methods and media, for the determination of resistance, have been developed [11,12,13,14,15,16,17,18,19,20,21].

### Objectives

The aim of this systematic review is to focus on the antibiotic susceptibility pattern of *L. pneumophila* isolates derived from clinical and/or water samples in accordance with (a) chronology, (b) geographical distribution, (c) the groups of antibiotics and (d) the methods used and the corresponding interpretation for the identification of microbial susceptibility.

## 2. Materials and Methods

### 2.1. Data Sources and Search Strategy

**Figure 1:**
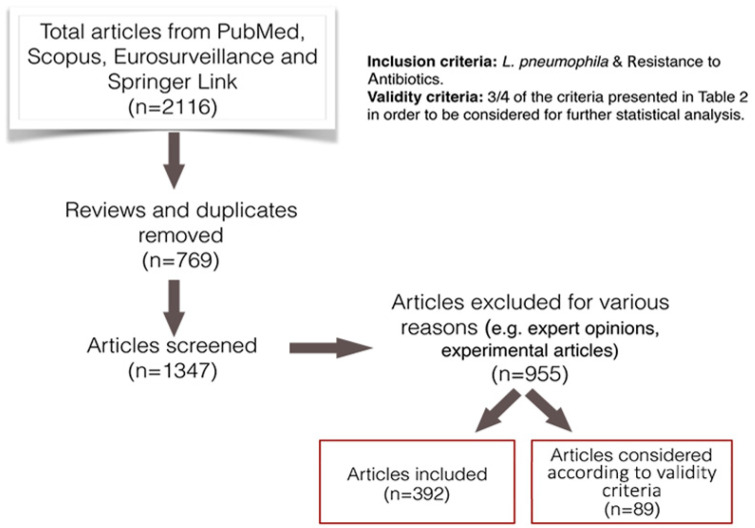
The flowchart (study selection process) was followed according to the PRISMA statement. The final 89 articles emerged according to the validity criteria.

The PRISMA statements were used in the search and the analysis process (Figure 1; [22,23]) PubMed, Scopus, the Eurosurveillance Journal and the Spingerlink electronic journal were the databases used to identify peer-reviewed papers associated with *L. pneumophila* and susceptibility to antibiotics. The database search was performed between October and December 2019. The search was performed using various key terms and their variations in combination using a Boolean search technique (Table 1). The titles, keywords and abstracts of the articles included in the online databases were searched for these search terms. Original articles, full or short communications and letters to the editor were included (reviews, conference abstracts, expert opinions and experimental articles were excluded). Only articles written in English were included. There were no restrictions on the geographical area or the year of publication.

### 2.2. Selection Criteria

Τhe terms “*Legionella pneumophila* and resistance to antibiotics” and “The origin of the isolates (water/clinical/biofilm)” were preset as “inclusion criteria” in the review. Papers meeting the inclusion criteria were evaluated according to pre-defined validity criteria (Table 2). Articles meeting at least 3/4 of the validity criteria were further considered for statistical analysis.

### 2.3. Data Extraction

Essential data were extracted from the studies meeting the inclusion and validity criteria. The data were formatted into a purpose-built Excel spreadsheet; the authors resolved any discrepancies. The data extraction was performed by four (4) of the authors; each author was responsible for one or two database searches, which were repeated twice for reasons of reproducibility (the same number of articles emerged). Additionally, articles in compliance with the set were evaluated by all of them for reasons of reliability. The data extracted from the eligible studies are presented in Table S1 in the raw data (Supplementary Materials).

### 2.4. Statistical Analysis

**Figure 2:**
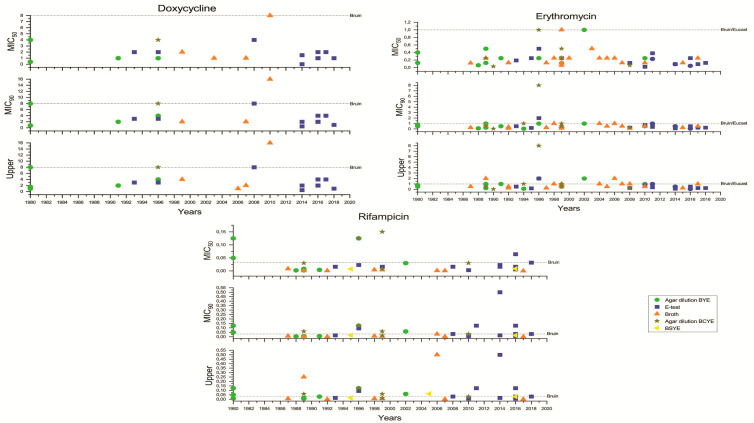
Distribution of MICs of ciprofloxacin, levofloxacin, azithromycin and clarithromycin based on different methodologies (Agar dilution on Buffered Yeast Extract (BYE), E-test, Broth dilution, Agar dilution on Buffered Yeast Charcoal Extract (BCYE), and Buffered Starch Yeast Extract (BSYE) plating). The epidemiological cut-off values proposed by Bruin et al. [29] and by the EUCAST (where available; 32) are shown as dotted horizontal lines.

**Figure 3:**
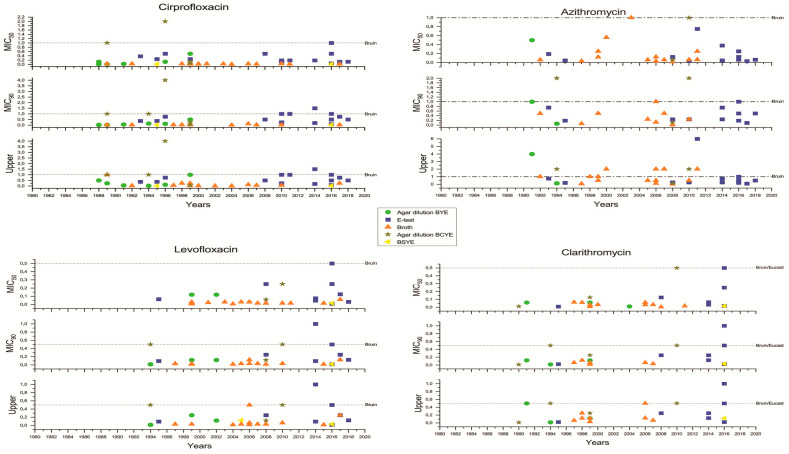
Distribution of MICs of docycycline, erythromycin and rifampicin based on different methodologies (Agar dilution on BYE, E-test, Broth dilution, Agar dilution on BCYE, and BSYE plating). The epidemiological cut-off values proposed by Bruin et al. [29] and by the EUCAST (where available; 32) are shown as dotted horizontal lines.

**Figure 4:**
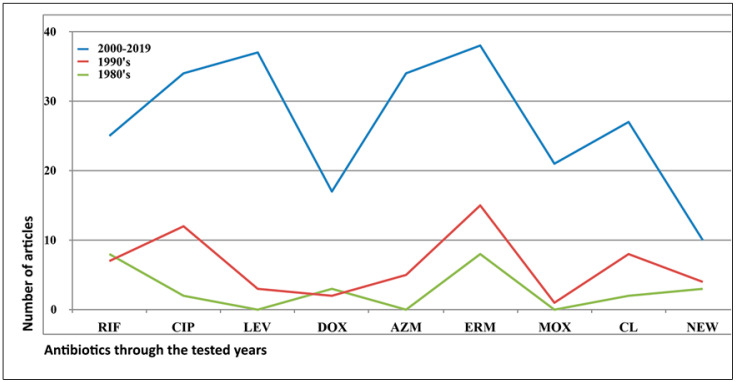
The use of antibiotics during the studied decades, 1980–2019. On the y axis, the number of articles in which each antibiotic has been mentioned is presented. Abbreviations: RIF, rifampicin; CIP, ciprofloxacin, LEV, levofloxacin; DOX, doxycycline; AZM, azithromycin; ERM, erythromycin; MOX, moxifloxacin; CL, clarithromycin; NEW, antimicrobial agents such as LBM415 and TP-271 (Table S1 raw data).

The analysis of the extracted data was performed using the standard Excel worksheets and the OriginLab 2020 software (v. 9.7.0.188; Copyright © 1991–2019 OriginLab Corporation, Arezzo, Italy). The graphs (Figure 2 and Figure 3) were based on the extracted data presented in Table S1, worksheet tables for graphics_2. Figure 4 was constructed using the extracted data as shown in Table S1, worksheet tables for graphics_1.Two articles were excluded because, although the authors mentioned that they did not find any decreased sensitivity against the antibiotics tested, they did not provide any specific values.

In deciding which antibiotics should be selected to work with, the following criteria were considered: (a) the most cited antibiotics in the revised articles included in the study (Table S1 raw data), (b) the guidelines of the Sanford Guide to Antimicrobial Therapy [24], and (c) the antibiotics administered in Greece [25,26,27,28]. This process resulted in the inclusion of seven (7) antibiotics in the search, these being ciprofloxacin (CIP), erythromycin (ERM), azithromycin (AZM), doxycycline (DOX), levofloxacin (LEV), rifampicin (RIF) and clarithromycin (CL).

As for the methods of assessment for microbial susceptibility, six main categories were identified: agar dilution, E-testing, plating, broth dilution, microdilution and disk testing (Table S1, worksheet tables for graphics_2). These were extracted, and in order to avoid the scattering of the data, microdilution was included in the Broth dilution group. The six main groups were further split into sub-groups based on the medium used [Buffered Yeast Extract (BYE), Buffered Yeast Charcoal Extract (BCYE), or Buffered Starch Yeast Extract (BSYE)] for Minimum Inhibitory Concentration (MIC) determination, resulting in the sub-categories E-testing on BYE, plating on BCYE, broth dilution on BCYE and Kirby Bauer on BCYE (Table S1, worksheet tables for graphics_2). Again, for the sake of graphical mutuality, the sub-categories, for which either values from a single study or only upper values were recorded, were excluded from further processing.

The time period when the included studies were carried out was between 1976 and 2019. The MIC_50_, MIC_90_ and upper values of the MIC ranges were processed in different worksheets and then categorized according to both the applied methodology and the year or period of years of each study. The “year” value was considered either the year of sample collection or, if the isolation was not mentioned, the year when each article was first submitted for publication or each article’s year of publication. In the case of time periods, we considered the “year” value as the last year of the collection period since none of the articles specify exact numbers of isolates per year. Since for the year 1976, a single study was included and only the values for the MIC ranges were given, for the sake of graphical mutuality, this year was merged with the studies carried out in the 1980s.

Two horizontal lines for *L. pneumophila* susceptibility testing were added to each graph demonstrating the Epidemiological Cut-Off values (ECOFF) values for each antibiotic. These were the ECOFF value guidelines proposed but not yet officially established by the European Society of Clinical Microbiology and Infectious Diseases (EUCAST) and the ones proposed by Bruin et al. [29], a reference used in many of the review papers. A recent publication by Sharaby [30] has also dealt with the important matter of the ECOFF lines. Sharaby [30] and Bruin et al. [29] suggest similar ECOFF lines; however, they differ from the ECOFF lines for the seven antibiotics included in the review, shown in Table 3. At present, the EUCAST has not set ECOFF values for all the antibiotics of interest here and that are widely used in the treatment of LD [31]. The EUCAST has approached the definition of MIC values for each antibiotic with the two-fold dilution series method [32]. During this procedure, the definition of where wild-type MICs end and in vitro-resistant isolates begin is based on a visual inspection of the histograms of the MICs for a single species [32,33]. This, however, depends on the number of observations; for some species, there is significant overlap of wild-type and resistant MICs and a lack of reproducibility [33].

## 3. Results and Discussion

### 3.1. The Process of the Selection of the Articles

The study selection process is presented in Figure 1. As a result, 89 published papers were finally considered in the statistical analysis of the review.

### 3.2. Geographical and Chronological Distribution of the Papers

Eighty-nine articles met the inclusion and the validation criteria and were further analyzed. The majority of the articles were reported during the period 2000–2019 (64%; 57/89), 23 articles were published in the 1990s (25%), and 11 articles were from the 1980s (11%). The oldest article identified in the present search was by Thornsberry et al. [34], who reported antibiotic-resistant *Legionella* isolates that originated from two major outbreaks, both of which occurred in the USA (Table S1 raw data). The oldest papers dealing with environmental samples are from 1992 (Table S1 raw data; [35,36]), even though in the literature, the first publication dealing with the isolation of Legionellae in water samples is dated much earlier [37]. The majority of the articles have been published by European research teams (40%; 36/89); 31 (35%) and 18 (20%) articles were published by USA and Asian researchers, respectively. There is one (1%) paper by a USA research team referring to European and American isolates, two articles (2%) by USA researchers including universal isolates and one (1%) by UK researchers including universal isolates (Table S1 raw data).

### 3.3. Type of Samples Included in the Studied Material

Sixty articles referred to clinical isolates (67.4%) and 18 articles reported water-related *L. pneumophila* isolates (20%) while 11 articles included both clinical and water isolates (12.4%). The above results are subject to the restriction that clinical articles were more abundant than environmental ones (60 vs. 18); this fact is a limitation within the research and, therefore, of this review.

#### 3.3.1. Clinical Samples

The type of specimen, in most cases being Broncho Alveolar Lavages/BAL and secretions from Respiratory Tract Infections/RTIs for the clinical isolates, was reported in 27 articles (45%; 27/60), and all of them were related to patients with CAP (Table S1 raw data). In most cases (65.2%; 58/89), the articles did not mention if the isolated *L. pneumophila* strains were related to CAP. There are fewer studies than expected considering the spread and the severity of the disease as well as the antibiotic scheme applied for therapy. In some papers dealing with both clinical and environmental strains, there is no discrimination between the two types of strains. One of the reasons for this discrepancy is that *L. pneumophila* usually causes a non-productive pneumonia, and it is difficult to obtain respiratory secretions for the isolation of the pathogen before the administration of any antibiotic [38]. Nevertheless, in one of these few studies, Miyashita N. et al. [39] evaluated the activities of various antibiotics against 58 clinical isolates and showed that levofloxacin, garenoxacin and rifampicin were the most potent drugs followed by ciprofloxacin, pazufloxacin, moxifloxacin, clarithromycin and azithromycin. The authors failed to reveal any differences between Serogroup 1 and Serogroups 2–15. Nevertheless, the authors still raise the issue of the intracellular activity of antibiotics, suggesting that in the case of *L. pneumophila*, the determination of the minimum extracellular concentration inhibiting intracellular multiplication could be necessary next to conventional MIC determinations.

#### 3.3.2. Water Samples

The site of sampling for the water-related isolates was reported in the 79.3% of the relevant articles (23/29, Table S1 raw data). The majority of the cases involved drinking water supplies (13 articles), while six (6) papers implicated drinking water networks along with cooling towers in the presence of *L. pneumophila* [40]. In most cases, articles regarding water isolates did not clearly indicate the microbial susceptibility of the isolates to the tested antibiotics; however, there were some studies from which we can extract some relevant information. In a study carried out in Italy [38], several environmental strains were tested against 10 antibiotics. The researchers recorded that all the *L. pneumophila* isolates were inhibited in the following decreasing order: doxycycline > tigecycline > cefotaxime. Moreover, the MICs of azithromycin, ciprofloxacin, levofloxacin, moxifloxacin and tigecycline were significantly lower for *L. pneumophila* Serogroups 2–15 than for *L. pneumophila* Serogroup 1. In another Italian study [41], the authors revealed that rifampicin was the most active antibiotic followed by levofloxacin, tigecycline and doxycycline. These results coincide with the results of previous studies [28,29,38,42,43,44]. Furthermore, all the *Legionella* strains were inhibited by low concentrations of macrolides and fluoroquinolones, while between the macrolides, clarithromycin was, overall, the most active drug compared to azithromycin. Again, differences in the MICs between *L. pneumophila* Serogroup 1 and Serogroups 2–15 were detected. Xiong et al. have reported the increased effectiveness of levofloxacin [45], followed by minocycline and doxycycline. The effectiveness of quinolones—ciprofloxacin, this time—was recorded in a later study by Sikora A. et al. [46]. Contrary to these findings, it was Sharaby et al. [30] who revealed some slight resistance against quinolones but an effectiveness of doxycycline, clarithromycin, rifampicin and trimethoprim sulphamethoxazole (SXT). Nevertheless, despite the finding of the potency of rifampicin, it should be noted that this antibiotic is not suggested for monotherapy due to the rapid appearance of resistance [47]. Similar findings and comments on the role of rifampicin were recorded in a study carried out in India [48] where tetracycline was also tested. However, it was, again, one of the less active agents. By contrast, Sharaby et al. [30] studied several environmental and clinical genotypes and revealed a discrepancy in antimicrobial agents’ susceptibilities, even in environmental strains that were isolated from adjacent points of the same water system. This is of importance considering that in some cases, the pneumonia caused by Legionella infection may hide a mixture of *L. pneumophila* strains [49,50].

### 3.4. Methods Used for the Determination of Resistance

Several methods have been proposed as suitable for the determination of MICs, such as the E-test, broth and agar dilution, and disk diffusion methods, in vivo and in vitro. Nonetheless, none of these methods is considered as a gold standard [29]. All 89 articles reported the method used for the identification of antibiotic susceptibility. In total, the most common method was the Broth Microdilution method, which has been proposed by the National Committee for Clinical Laboratory Standards [51] (later renamed the Clinical Laboratory Standard Institute [52]). The Broth Microdilution method was used in 37/89 studies (41.6%), and the interpretation criteria were used accordingly (Table S1 raw data). There was only one paper, Jia et al. [53], which used the Broth Microdilution method with the ECOFF interpretation criteria [54]. Classifying the results according to year or a period of years, it was observed that a significant number of papers from the 1980s and 1990s (18/37; 48.6%) used the Broth Microdilution method according to previously published protocols, such as Edelstein et al. [55,56,57,58], Hoogkamp-Korstanje [59] and Jones et al. [60] (Table S1 raw data; Figure 2 and Figure 3). Looking at the studies published in the time period 2000–2019, it is interesting to note that 17/57 articles used the Broth Microdilution method, as recommended by the CLSI, but then applied methods cited by older publications instead of the CLSI-recommended method for the interpretation of the results. For example, Blasco et al. [61] cited Saito et al. [62], Jonas et al. [63] cited Edelstein et al. [56], Kunishima et al. [64] cited Liebers et al. [65], Mallegol et al. [66] cited Stout et al. [67], and Tan et al. [68] cited Stout et al. [69] (Table S1 raw data). The E-test method was the second most cited (19/89; 23.3%), with a slight difference from the Agar Dilution method (15/89; 21%) (Figure 2 and Figure 3). It seems that the most recent publications (2000–2019) used the E-test method proposed by the EUCAST for the interpretation criteria (10/19 articles), yet there were still some articles using older citations for the applied method, such as Alexandropoulou et al. (2013 and 2019) [25,26], Lai et al. (2010) [70] and Shadoub et al. (2015) [71] (Table S1 raw data). None of the abovementioned articles (2000–2019) explained why they relied on using publications rather than the guidelines from the CLSI or EUCAST. It is not clear why the research groups prefer to apply media and methods suggested by earlier publications and not those suggested by international standardization organizations such as the CLSI and EUCAST. As a result, there does not seem to be any standardization in the application of the methods over time. This is particularly true in the studies of the 1980/1990s where the Agar Dilution method (15/89; 16.9%) seems to have been more popular. During this period, five articles (5/15) published in the 1980s applied either “in house” protocols developed by the authors (Table S1 raw data; [55,72,73]) or protocols based on previous publications [Table S1 raw data; 34]. Of the five articles (5/15) in the 1990s, only one applied the National Committee for Clinical Laboratory Standards (NCCLS) method (Table S1 raw data; [74]), two papers used older citations (Table S1 raw data; [27,75]) and two did not mention the reference method at all (Table S1 raw data; [76,77]). Finally, there were five studies from the 2000–2019 period that applied the Agar Dilution methods, mainly using the NCCLS and CLSI protocols (Table S1 raw data; [78,79,80,81], and only one article used a very old published study (Table S1 raw data; [39]). In 8/89 (9%) of the articles, the authors used the Kirby–Bauer method and followed the protocol proposed by the CLSI (Table S1 raw data; [82,83]) or older citations (Table S1 raw data; [40,84]). Finally, there were 9/89 (10%) articles that applied a combination of the methods (Table S1 raw data; [42,46,56,65,85,86,87,88,89]) and one multi-center study from Dunbar et al. 2007 [90]. In the Dunbar et al. [90] study, they performed an alternative Broth Microdilution method (Sensititre microtitre plates containing freeze-dried antimicrobial agents) in order to evaluate the activity of telithromycin against a worldwide *L. pneumophila* isolate set. From the above-extracted data, it can be concluded that—until recently, when a protocol was proposed by the EUCAST [54]—the lack of a common protocol for the testing of *L. pneumophila’s* microbial resistance, as well as common criteria for interpreting the results (e.g., ECOFF), has led many researchers to select whatever published protocol is appropriate for each study. Due to this variety in the application of the methods, it is difficult to compare and correlate the published data. This serves to enhance the difficulty of studying the microbial susceptibility of *L. pneumophila*. This limitation has been cited in the published literature. For example, a study was carried out by Sharaby et al. [30], where it was reported that quinolones performed better than macrolides, resulting in a reduced length of stay and decreased time until clinical improvement. However, the authors went on to emphasize that such findings could be a result of the different methods used for susceptibility testing and should always be treated with caution.

### 3.5. Media Used for the Identification of L. pneumophila’s Antimicrobial Susceptibility

In 80 articles, only one medium was used for the identification of *L. pneumophila’s* antimicrobial susceptibility. However, in seven articles, two or three different culture media were used in order (a) to compare the results (Table S1 raw data; [65,74,90]), (b) to evaluate the addition of charcoal in the medium (Table S1 raw data; [86]), and (c) when two different methods were applied (Table S1 raw data; [89]). The most common medium used was BCYE with or without α-ketoglutarate (BCYE in 23 articles and BCYE-α in 26 articles), which is proposed by the CLSI and the EUCAST [52,54]. The culture media BSYE (five articles), BYE (eight articles) and BYE-a (10 articles) were also used with minor differences compared to the proposed medium. Finally, Mueller–Hinton agar was used exclusively when Kirby–Bauer was applied as the reference protocol (Table S1 raw data). The use of different media played its own role in the resulting MICs. According to the EUCAST, different methods, particularly with different media, yield different MICs. The use of media containing charcoal showed that the addition of charcoal resulted in elevated MICs for most of the relevant agents [54]. It is crucial for the laboratories studying the microbial resistance of the bacterium for routine work to use the proposed, most reliable medium. As far as we know, the EUCAST has not still established ECOFF values for all the antibiotics commonly used; however, the published MIC distributions give an indication of the MICs for wild-type isolates using gradient tests on the recommended medium (available at https://mic.eucast.org/Eucast2/SearchController/search.jsp?action=performSearch&BeginIndex=0&Micdif=mic&NumberIndex=50&Antib=1&Specium=230). The difficulties in determining the MICs for clinical strains have been well explained. This arises from the fact that the acquisition of a suitable specimen from a patient before the administration of antibiotics is not always feasible [38]. Combined with this, there is always the issue of antibiotic inactivation (examples are tetracyclines, fluoroquinolones, and macrolides) due to the presence of charcoal in the medium, which is necessary for the proliferation of *Legionella* [28,29,42,43,46,47]. Moreover, the results obtained from each study should be interpreted with caution since in certain circumstances, the in vitro results may poorly correlate with the clinical effectiveness [42] of each antibiotic. To further support this, the EUCAST has published an updated guideline on MIC determination for *L. pneumophila.* However, as the committee states, it should be used as guidance for antibiotic resistance rather than for the guidance of treatment [54].

### 3.6. Groups of Antibiotics and Antimicrobial Agents

The antibiotics and antimicrobial agents used for the study of *L. pneumophila*’s antimicrobial sensitivity from all the reported methods are presented in Supplementary Table S1 (Table S1 worksheet_abbreviations of antibiotics). In total, 89 different antibiotics and antimicrobial agents were reported; rifampicin (RIF), ciprofloxacin (CIP), levofloxacin (LEV), doxycycline (DOX), azithromycin (AZM), erythromycin (ERM), moxifloxacin (MOX) and clarithromycin (CL) were most commonly cited. Erythromycin seems to be the most commonly used of the antibiotics, with 60 papers referring to it. The next most frequently cited are ciprofloxacin (50 papers), levofloxacin (40 papers), azithromycin (39 papers), rifampicin (37 papers), clarithromycin (36 papers) and doxycycline (23 papers) (Table S1 raw data). This result is not surprising, as when the Legionellosis area started in the 1970s, the recommended antibiotic and, therefore, the most widely used was erythromycin, which belongs to the group of macrolides (Figure 4). Erythromycin was first introduced in 1952, and since then, it has been used widely against many infections of the respiratory system. Later, in the 1980s, another macrolide, azithromycin, was introduced and has been used to replace erythromycin in many cases (Figure 4). Ciprofloxacin, belonging to the quinolones group, was also introduced almost simultaneously (Figure 4). For Legionellosis, macrolides (AZM) and quinolones (CIP, LEV, MOX, GEM and TROV) are recommended as the antibiotics of choice [24] (Figure 4). A decrease in the sensitivity to antibiotics was identified in approximately half of the tested articles (46%; 41/89), with the antibiotics erythromycin, ciprofloxacin, azithromycin and clarithromycin being reported most often. In 15/89 of the articles, the isolates are reported as sensitive to all the tested antibiotics (17%) (Table S1 raw data). On the other hand, the percentage of the non-defining-resistance articles was high enough (33/89; 37%). This has been identified as one of the limitations of this review. Half of these studies did not use specific interpretation criteria for defining the microbial susceptibility of the *L. pneumophila* isolates (19/33; 57.6%) (Table S1 raw data), which is consistent with the lack of a universal protocol for the testing of *L. pneumophila* microbial susceptibility. This was especially true for the articles published in the 1980s and 1990s (Table S1 raw data). However, even in studies where the CLSI or EUCAST criteria were applied, the determination of antibiotic susceptibility was not defined (Table S1 raw data; [12,67,91,92,93,94,95,96,97,98,99]. In most articles, the authors preferred to comment on the activity of the tested antibiotics against the studied isolates rather than define the susceptibility against them (Table S1 raw data; [96,100]. The lack of a clear characterization of the *L. pneumophila* isolates as resistant or susceptible in the proposed CLSI and EUCAST protocols adds further confusion to the study of *L. pneumophila* microbial susceptibility. This fact seems to be an important public health issue as it is well established that *Legionella* accounts for approximately 1 to 10 percent of cases of CAP (Table S1 raw data; [29,30,70,90,93,100,101,102]. For most patients with CAP, the etiology is not known at the time of diagnosis or when empiric treatment is applied [103].

### 3.7. Criteria Used for the Choice of the Antibiotic Scheme for the Patient

How do laboratories decide on the antibiotics they are going to test for resistance in *L. pneumophila*? Historically—since the discovery of the progenitor macrolide, erythromycin, in 1950—many derivatives have been synthesized. This has led to compounds with better bioavailability and acid stability and improved pharmacokinetics. These efforts led to the second generation of macrolides, including well-known members such as azithromycin and clarithromycin. Subsequently, to address increasing antibiotic resistance, a third generation of macrolides displaying improved activity against many macrolide resistant strains was developed. However, these improvements were accompanied by serious side effects, leading to disappointment and causing many researchers to stop working on macrolide derivatives once treatments had reached the end [104]. Chronic treatment with macrolides is associated with adverse effects including gastrointestinal symptoms, interactions with other drugs and cardiovascular complications. Of the macrolides, azithromycin is associated with the lowest interactions and adverse effects and is also the most investigated [105]. Azithromycin use was not associated with a higher risk of death, particularly in younger population; nevertheless, older populations might be at higher risk of death with the current use of azithromycin, and an alternative therapy should probably be considered [106]. According to the above-published information, it seems that the laboratory testing of *Legionella’s* microbial susceptibility follows the routine clinical practice as prescribed by each patient’s doctor. As a result, they do not test for susceptibility according to standard and approved protocols. The selection of the antibiotics tested according to the provenance of the isolates is of interest. As for resistance testing in isolates from water samples, ciprofloxacin seems to be the first choice (13/18 articles; 72%), followed by erythromycin (66%), azithromycin (55%) and rifampicin (55%). Levofloxacin and clarithromycin were both reported in nine articles (50%), and doxycycline, in seven (39%) (Table S1 worksheet sample vs antibiotics). In articles dealing with clinical isolates, the same antibiotics were reported, with erythromycin being the most common (39 articles; 65%), followed by ciprofloxacin (29; 48%), levofloxacin (26; 43%), azithromycin and rifampicin (24 and 23 papers, respectively; 40%), clarithromycin (23; 38%) and doxycycline (12; 20%) (Table S1 worksheet geographical distribution).

Finally, in studies where clinical and environmental isolates were examined, a combination of antibiotics was observed. Erythromycin and rifampicin were most frequently reported (nine and eight articles, respectively; 81%) (Table S1, worksheet sample vs. antibiotics). In conclusion, it seems that there is no correlation between the appearance of a specific antibiotic and the type of sample.

### 3.8. The Choice of Antibiotics and Geographical Distribution

As for the geographical distribution of the use of antibiotics, in Europe, ciprofloxacin seems to be the antibiotic of choice (27 articles; 77%). Meanwhile, erythromycin was most commonly cited in articles from the USA (27; 87%) and Asia (16; 88%) (Table S1, worksheet tables for graphics_1). There does not seem to be any correlation between the use of an antibiotic and the geographical area. From the extracted data, it seems that there is no common guideline proposing a specific antibiotic panel for the testing of *L. pneumophila* microbial susceptibility. In all 89 articles, the choice of the antibiotic panel depended on the protocol (NCCLS, CLSI, EUCAST or previous publications) that was applied by each research group.

## 4. Conclusions

The antibiotic of choice for treatment and, therefore, testing for resistance has considerably changed over time. Nevertheless, the consensus is that quinolones (ciprofloxacin and levofloxacin) and macrolides (clarithromycin and azithromycin) have potent antimicrobial activity against *L*. *pneumophila*. As for the choice of treatment, it should always be kept in mind that whatever the in vitro susceptibility determined, no clear-cut correlation with the clinical outcome exists. As a result, more effort should be spent on the determination of MICs and, therefore, the specific ECOFF values. Infections with multiple strains seem to be a common problem; therefore, greater emphasis on determining the isolates’ genotypes seems to be necessary, as the application of antimicrobial combinations may be necessary in these cases. Extensive monitoring of *Legionella* isolates needs to be performed, especially in man-made water systems, to recognize early changes in the antibiotic resistance pattern and to prevent impacts on clinical isolates.

The above conclusions need to be evaluated in light of the limitations of the data, such as the relatively high percentage of the non-defining-resistance articles; the papers dealing with clinical isolates outnumbering the ones dealing with water samples; and, finally, the large difference in the available information between the tested time periods.

## Figures and Tables

**Table 1:** The key terms and their variations in combination using Boolean search technique.

*Legionella pneumophila* OR *L. pneumophila* AND Resistance to antibiotics
*Legionella pneumophila* OR *L. pneumophila* AND Antibiotic susceptibility
*Legionella pneumophila* OR *L. pneumophila* AND Antibiotic sensitivity
*Legionella pneumophila* OR *L. pneumophila* AND cooling towers AND Resistance to antibiotics
*Legionella pneumophila* OR *L. pneumophila* AND drinking water AND Resistance to antibiotics
*Legionella pneumophila* OR *L. pneumophila* AND swimming pool water AND Resistance to antibiotics
*Legionella pneumophila* OR *L. pneumophila* AND bath water AND Resistance to antibiotics
*Legionella pneumophila* OR *L. pneumophila* AND clinical AND Resistance to antibiotics
*Legionella pneumophila* OR *L. pneumophila* AND cooling towers AND Resistance to antibiotics AND biofilm
*Legionella pneumophila* AND swimming pool water AND Resistance to antibiotics AND biofilm
*Legionella pneumophila* AND bath water AND Resistance to antibiotics AND biofilm
*Legionella pneumophila* OR *L. pneumophila* AND clinical AND Resistance to antibiotics AND biofilm
*Legionella pneumophila* AND Community Acquired Pneumoniae AND Resistance to antibiotics
*Legionella pneumophila* AND CAP AND Resistance to antibiotics

**Table 2:** The validity criteria developed a priori.

Validity Criteria
The site of sampling for the water and biofilm isolates (cooling towers, drinking water, swimming pool water, bath water)
The specimen(s) for the clinical isolates
The method chosen for antimicrobial testing
The interpretation criteria used for the evaluation of the antimicrobial sensitivity

**Table 3:** Epidemiological Cut-Off (ECOFF) values proposed by European Society of Clinical Microbiology and Infectious Diseases (EUCAST) [32]; Bruin et al. (2012) [29] and Sharaby et al. (2019) [30].

Antibiotics	ECOFFs (mg/L)		
	Bruin, 2012	Sharaby, 2019	EUCAST, 2017
CIP	1	4	ND
ERM	1	0.5	1
AZM	1	2	ND
LEV	0.5	1	ND
CL	0.5	0.5	0.5
DOX	8		
RIF	0.032	0.023	ND

Abbreviations: ciprofloxacin (CIP), erythromycin (ERM), azithromycin (AZM), doxycycline (DOX), levofloxacin (LEV), rifampicin (RIF) and clarithromycin (CL); ND: non-detected.

## Supplementary Materials

The following are available online at https://www.mdpi.com/1660-4601/17/16/5809/s1. Table S1 raw data; abbreviations of antibiotics; tables for graphics 1; tables for graphics 2; sample vs antibiotics; geographical distribution.
